# Breast Cancer Status in Iran: Statistical Analysis of 3010 Cases between 1998 and 2014

**DOI:** 10.1155/2017/2481021

**Published:** 2017-11-01

**Authors:** Mohammad Esmaeil Akbari, Soheila Sayad, Saed Sayad, Maryam Khayamzadeh, Leila Shojaee, Zeynab Shormeji, Mojtaba Amiri

**Affiliations:** ^1^Cancer Research Center, Shahid Beheshti University of Medical Sciences, Tehran, Iran; ^2^Department of Surgery, Firoozgar Clinical Research Development Center, Iran University of Medical Sciences, Tehran, Iran; ^3^Department of Chemical Engineering and Applied Chemistry, Data Mining Research Group, University of Toronto, Toronto, ON, Canada; ^4^Faculty of Management and Medical Information, Iran University of Medical Sciences, Tehran, Iran; ^5^Chemical Injuries Research Center, Baqiyatallah University of Medical Sciences, Tehran, Iran

## Abstract

**Background:**

Breast cancer is the 5th leading cause of cancer death in Iranian women. This study analyzed 3010 women with breast cancer that had been referred to a cancer research center in Tehran between 1998 and 2014.

**Methods:**

In this retrospective study, we analyzed 3010 breast cancer cases with 32 clinical and paraclinical attributes. We checked the data quality rigorously and removed any invalid values or records. The method was data mining (problem definition, data preparation, data exploration, modeling, evaluation, and deployment). However, only the descriptive analyses' results of the variables are presented in this article. To our knowledge, this is the most comprehensive study on breast cancer status in Iran.

**Results:**

A typical Iranian breast cancer patient has been a 40–50-year-old married woman with two children, who has a high school diploma and no history of abortion, smoking, or diabetes. Most patients were estrogen and progesterone receptor positive, human epidermal growth factor (HER) negative, and P53 negative. Most cases were detected in stage 2 with intermediate grade.

**Conclusion:**

This study revealed original findings which can be used in national policymaking to find the best early detection method and improve the care quality and breast cancer prevention in Iran.

## 1. Background

Breast cancer is the most common cancer in women worldwide, with nearly 1.7 million new cases diagnosed in 2012 (second most common cancer overall). This represents about 12% of all new cancer cases and 25% of all cancers in women [1, 2]. Since many published works on breast cancer are from North America, Europe, and Japan, we tend to consider breast cancer primarily from their points of view. But this cancer is becoming a bigger challenge for developing countries.

In Iran, breast cancer is one of the most frequent malignancies in women. Its peak incidence age in Iranian women is in the fourth and fifth decades of life, which is a decade younger than the global peak age of incidence [3, 4]. Because of Iran's socioeconomic status and the important role of women as mothers and the main caretakers of an extended family, breast cancer significantly damages the patient's family [5]. This makes planning and policymaking for early diagnosis of this disease and reducing its complications and mortality an important public health sector priority in Iran.

There have been a limited number of epidemiological studies on breast cancer in Iran, but none has been conducted on its early diagnosis [3]. Therefore, there has been an obvious and urgent need for an accurate statistical analysis of breast cancer in Iran not only to explore, understand, and explain this cancer more accurately but to develop a robust early diagnosis platform for public health professionals.

This study set out to analyze 3010 breast cancer cases (all women) that had referred to a Cancer Research Center in Tehran between 1998 and 2014.

## 2. Materials and Methods

This study's data were obtained from the Cancer Research Center of Shahid Beheshti University of Medical Sciences in Tehran. The Cancer Research Center's team has been collecting clinical, pathological, biological, and demographic information on breast cancer patients since 1998. The Cancer Research Center is a major referral center for breast cancer patients. It has cases from all provinces of Iran. It has the biggest data records of breast cancer cases in the country and can be representative of all Iran.

In total, we gathered, cleaned, and analyzed data from 3010 breast cancer patients. All clinical and paraclinical data have been obtained by two surgeons and recorded by a medical information specialist. The data quality has been checked rigorously and any invalid values or records have been excluded from the study. We saved the data in a relational database and followed and implemented data mining methodology (problem definition, data preparation, data exploration, modeling, model evaluation, and deployment) for long-term maintenance and accessibility for current and future statistical analysis and predictive modeling (e.g., score cards and survival analysis). We transferred our data to a SQL Server database and did all necessary quality checks. Then, we explored data using descriptive (univariate) and bivariate analysis using R. However, here we have presented the descriptive analyses' results of all 32 variables regarding breast cancer in Iran.

## 3. Results

**Figure 1:**
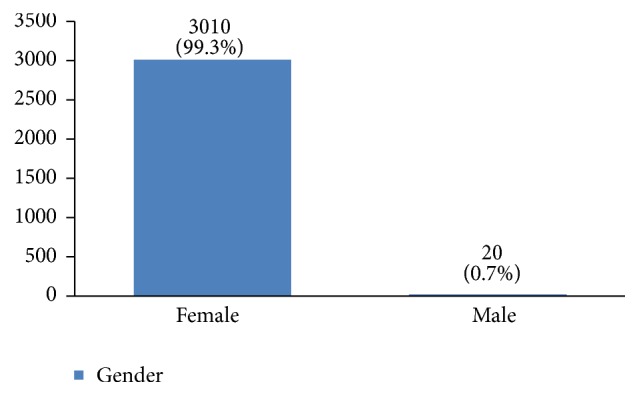
The sex distribution of the studied breast cancer patients.

A retrospective analysis was done on breast cancer patients diagnosed between 1998 and 2014 at the Cancer Research Center of Shahid Beheshti University of Medical Sciences in Tehran. Thus, the profiles of 3010 women with breast cancer were evaluated in this study. All the studied patients were followed up in the past 20 years. We excluded all men from our analyses (Figure 1). A total number of 32 variables were data mined and underwent descriptive analysis.

### 3.1. Demographics

**Figure 2:**
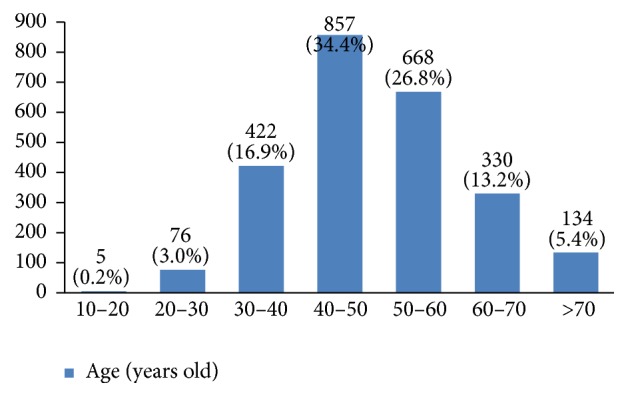
The age groups of the studied breast cancer cases.

**Figure 3:**
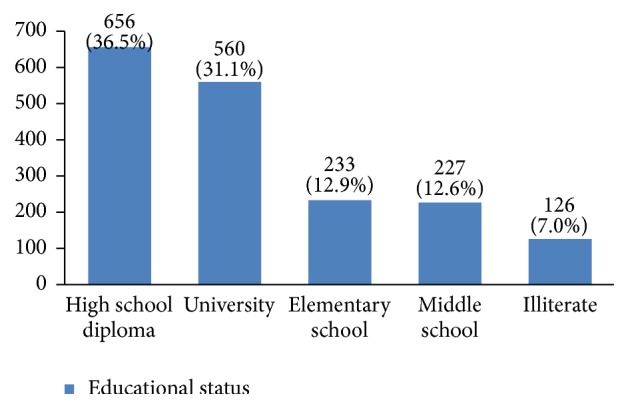
The educational status of the studied breast cancer cases.

**Figure 4:**
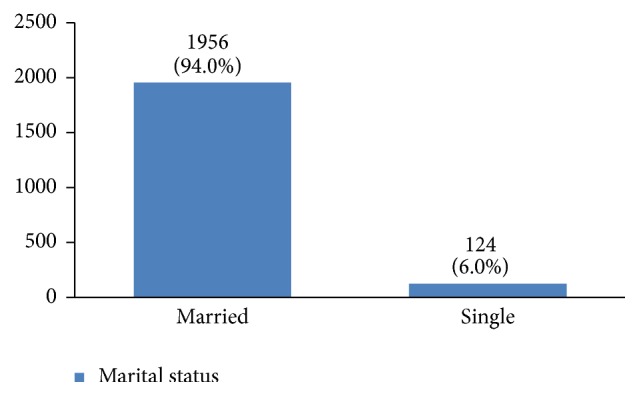
The marital status of the studied breast cancer cases.

The profiles of 3010 women with breast cancer were evaluated in this study. Their average age was 49.1 ± 11.6 years old. Most of them were between 40 and 50 years old (Figure 2). 36% of them had a high school degree, 31% university degree, and 26% elementary and middle school degree and only 7% were illiterate (Figure 3). 94% of the studied women were married (Figure 4).

### 3.2. Past History

**Figure 5:**
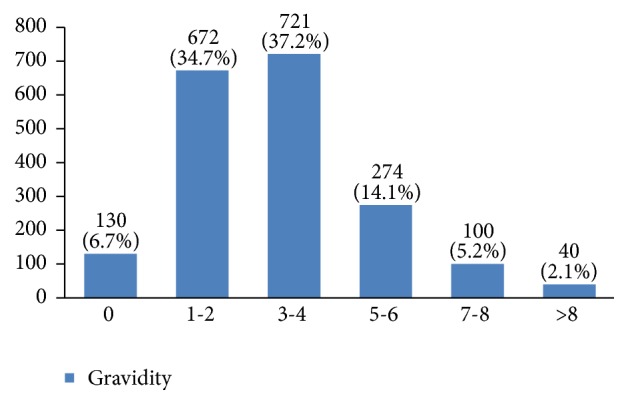
The gravidity of the studied breast cancer cases.

**Figure 6:**
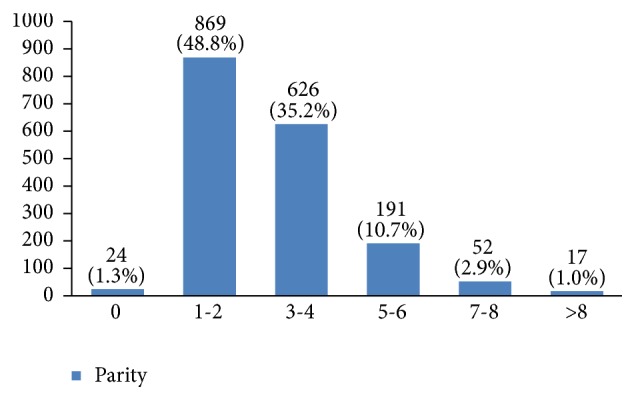
The parity of the studied breast cancer cases.

**Figure 7:**
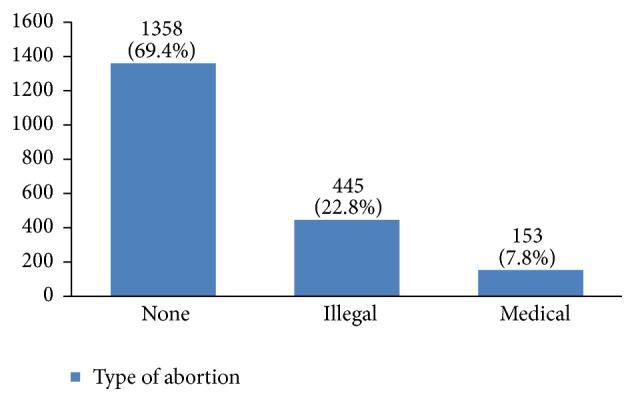
The abortion type of the studied breast cancer cases.

**Figure 8:**
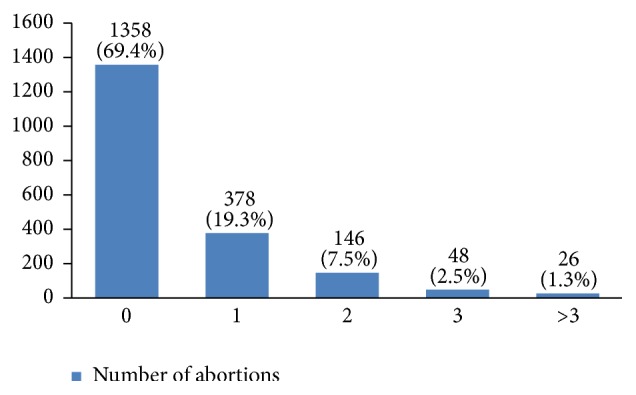
The number of abortions of the studied breast cancer cases.

**Figure 9:**
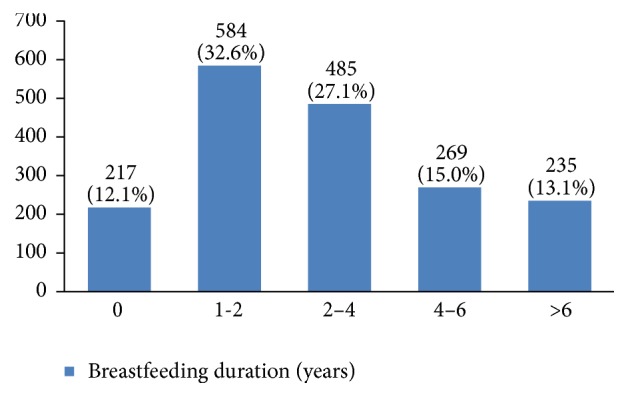
The breast feeding duration of the studied breast cancer cases.

**Figure 10:**
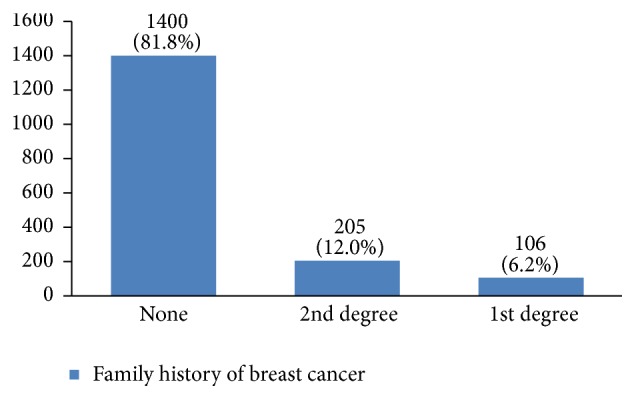
The family history of the studied breast cancer cases.

**Figure 11:**
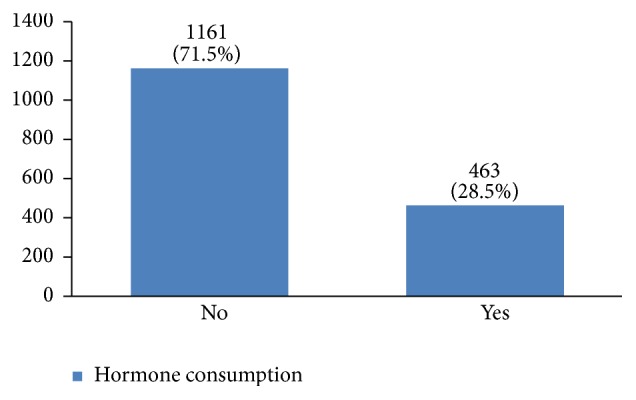
The hormone consumption of the studied breast cancer cases.

**Figure 12:**
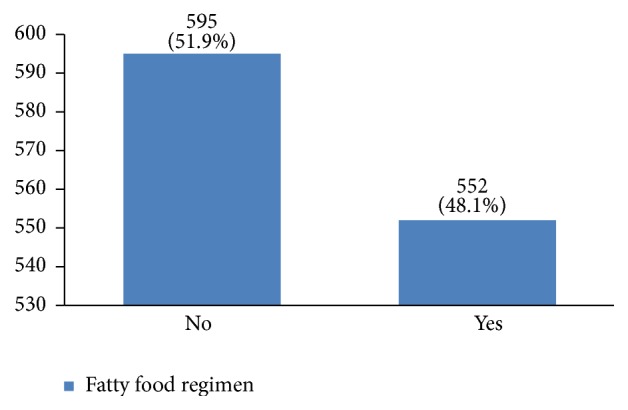
The fatty food regimen of the studied breast cancer cases.

**Figure 13:**
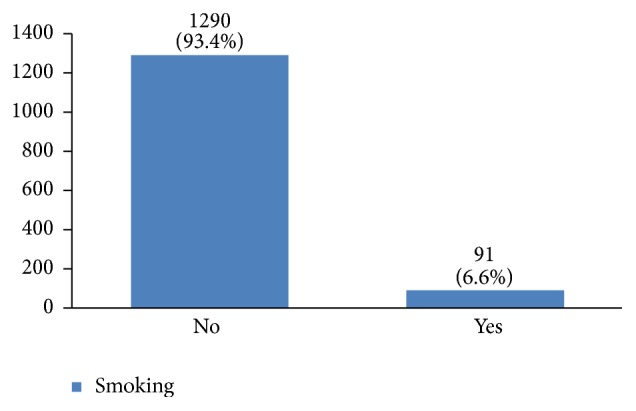
The smoking habit of the studied breast cancer cases.

**Figure 14:**
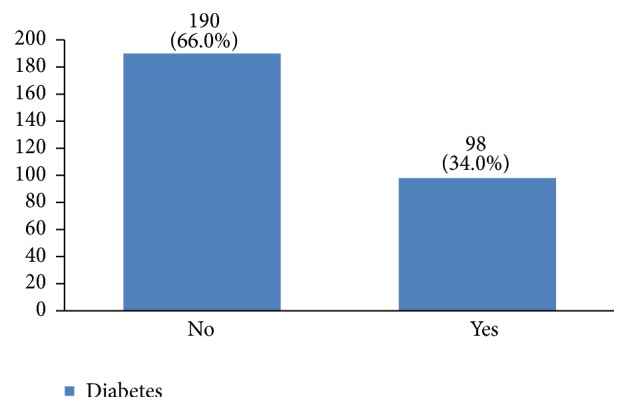
The diabetes status of the studied breast cancer cases.

**Figure 15:**
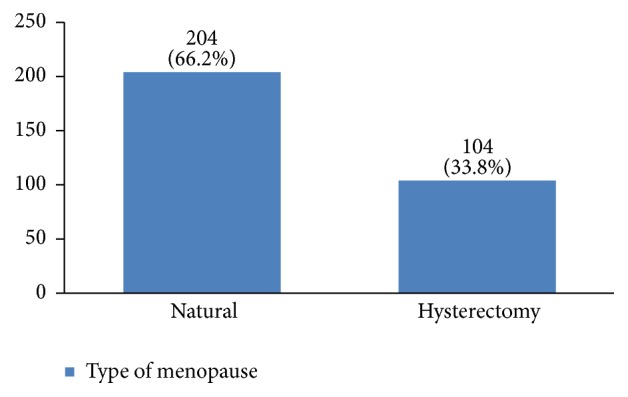
The menopause type of the studied breast cancer cases.

**Figure 16:**
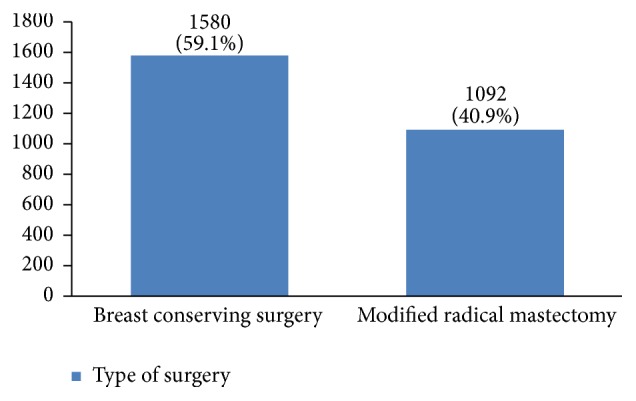
The surgery type of the studied breast cancer cases.

In our study, most cases (72%) reported 1 to 4 gravidity and 50% had 2 or less parity (Figures 5 and 6). Moreover, 11.5% of the patients had no pregnancies and 12.6% had no labor experience. 30% of the patients had one (19%) or more abortions (11%), mostly illegal (Figures 7 and 8). 12% had no breastfeeding, 33% breastfed for up to two years, and 27% breastfed for up to four years (Figure 9). Surprisingly, more than 80% of the studied women had reported no known family history of breast cancer (Figure 10). Only 28% of the patients had a history of using hormones (estrogen and progesterone) (Figure 11). 48% of the patients had a high-fat regime (Figure 12), only 7% were smokers (Figure 13), and 34% had diabetes (Figure 14). Two-thirds of the patients experienced menopause naturally and one-third had secondary menopause due to hysterectomy (Figure 15). Finally, 41% of the patients had undergone breast conserving surgery and 59% had done modified radical mastectomy (Figure 16).

### 3.3. Histopathology

**Figure 17:**
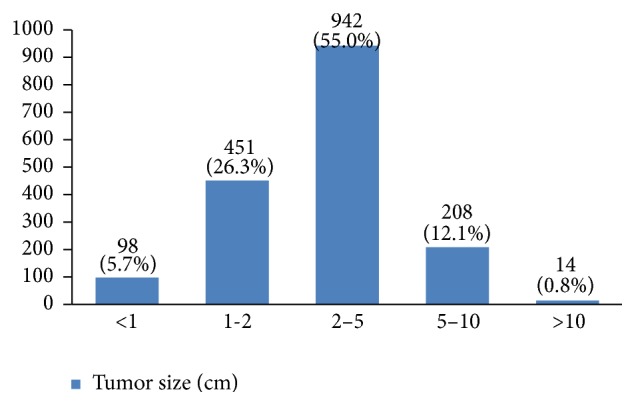
The tumor size of the studied breast cancer cases.

**Figure 18:**
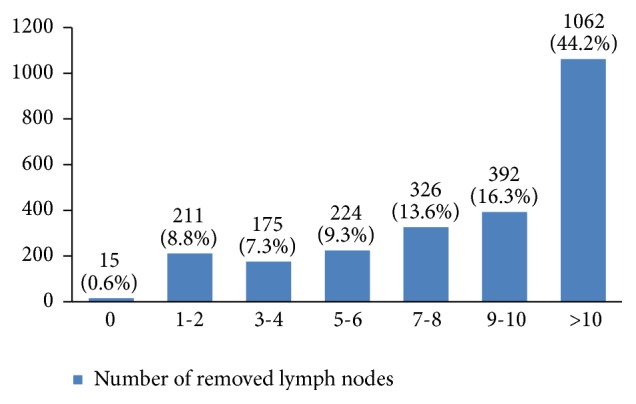
The number of removed lymph nodes in the studied cases.

**Figure 19:**
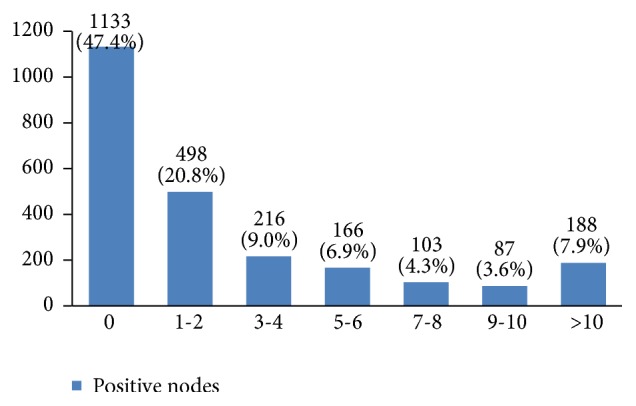
The number of pathologically positive nodes in the studied cases.

**Figure 20:**
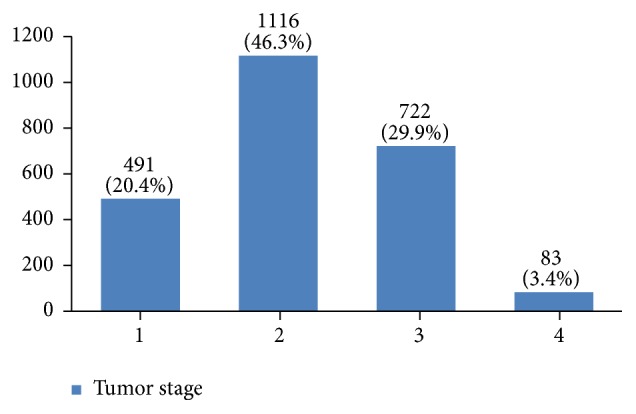
The tumor stage of the studied breast cancer cases.

In almost 55% of the patients the tumor size was between 2 and 5 centimeters (Figure 17). In many patients (44%) more than 10 lymph nodes had been removed (Figure 18). However, the highest rate of pathologically positive nodes (21%) was between 1 and 2 (Figure 19). Regarding tumor stage, 20% of the studied women were in stage I, 46% in stage II, 30% in stage III, and 4% in stage IV (Figure 20).

**Figure 21:**
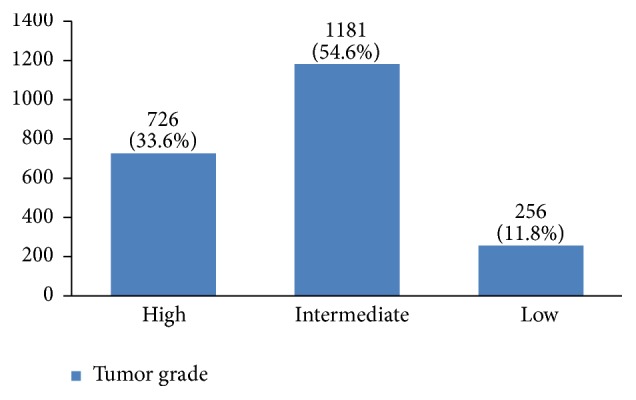
The tumor grade of the studied breast cancer cases.

**Figure 22:**
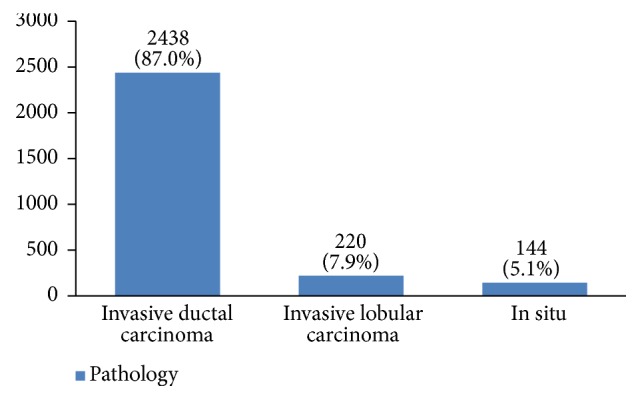
The pathology of the studied breast cancer cases.

**Figure 23:**
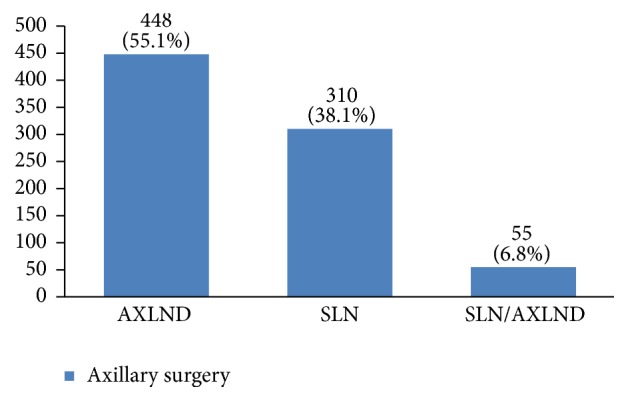
The axillary surgery type of the studied breast cancer cases.

**Figure 24:**
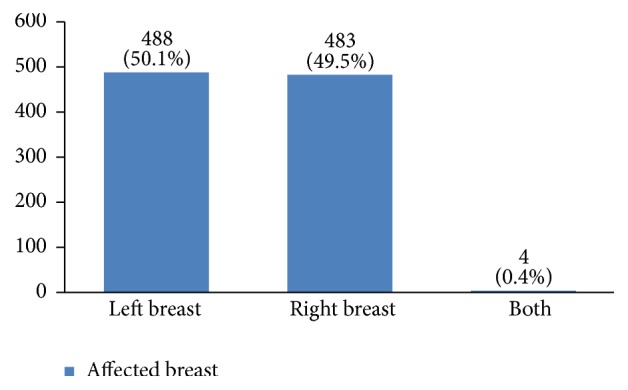
The involved breast sides (left or right) in the studied cases.

**Figure 25:**
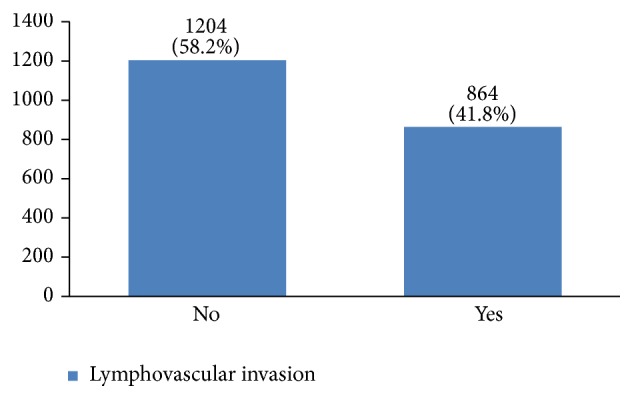
The percentage of lymphovascular invasion in the studied cases.

**Figure 26:**
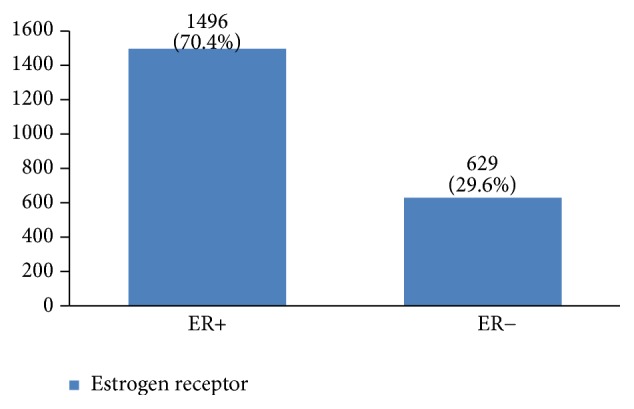
The percentage of estrogen receptor in the studied cases.

**Figure 27:**
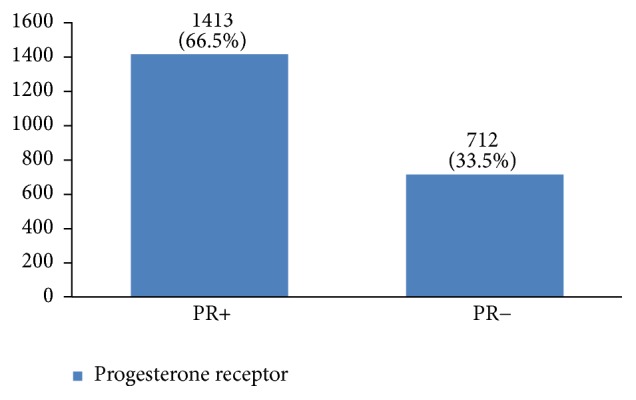
The percentage of progesterone receptor in the studied cases.

Regarding tumor grade, 54% were in intermittent grade, 34% in high grade, and 12% in low grade (Figure 21). Concerning pathology, 87% had invasive ductal carcinoma, 8% had invasive lobular carcinoma, and 5% were in situ (Figure 22). Regarding the type of axillary surgery, 55% had undergone axillary dissection, 38% sentinel node biopsy, and 7% sentinel node biopsy plus axillary dissection (Figure 23). In most patients both sides of their bodies (right and left breasts) were involved equally and there were just a few cases with cancer on both sides (Figure 24). 42% of the patients had lymphatic vascular invasion (Figure 25). Positive estrogen receptor (ER+) was reported in 70% of cases compared to 66% who had positive progesterone receptor (PR+) (Figures 26 and 27).

**Figure 28:**
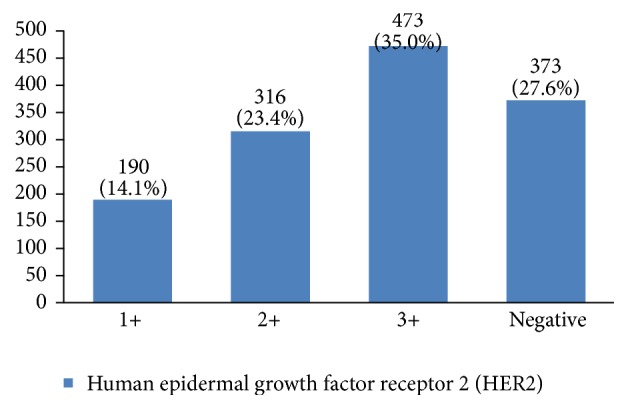
The distribution of HER2 types in the studied cases.

**Figure 29:**
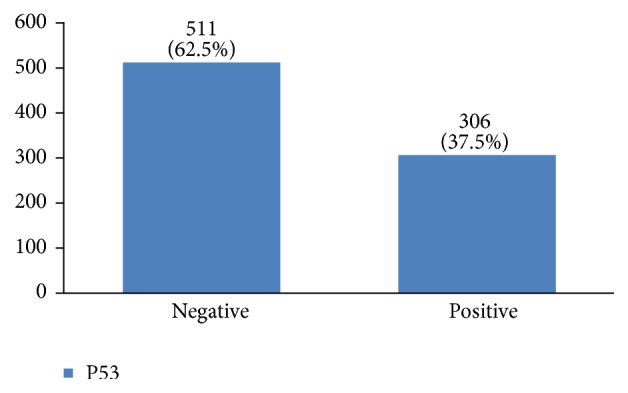
The distribution of P53 in the studied cases.

**Figure 30:**
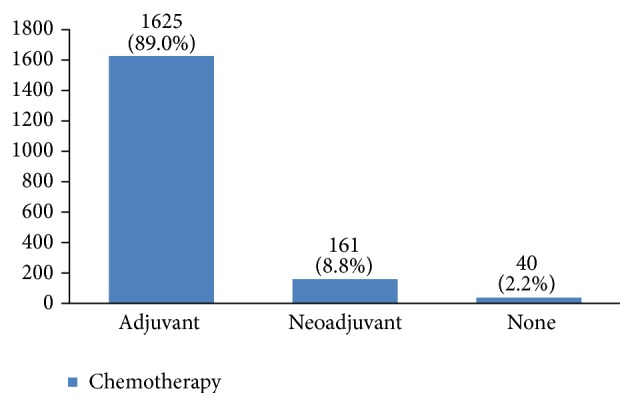
The chemotherapy type in the studied cases.

**Figure 31:**
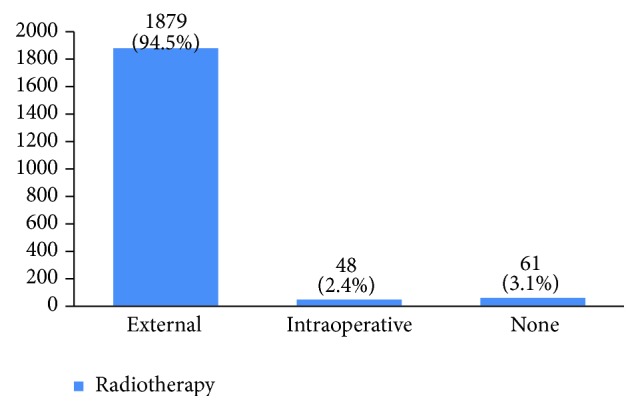
The radiotherapy type in the studied cases.

**Figure 32:**
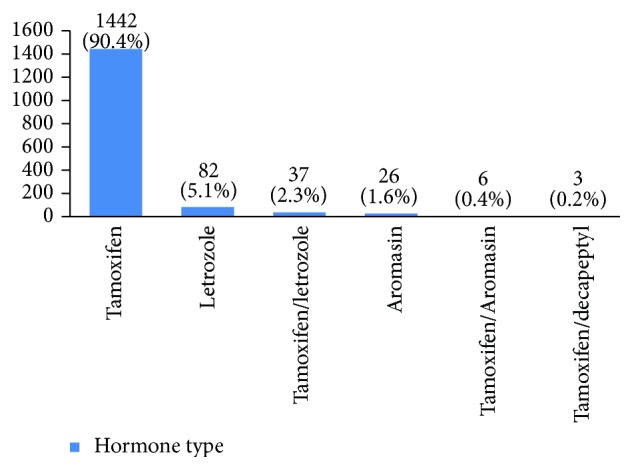
The hormone type of the studied cases.

Furthermore, 28% of cases were human epidermal growth factor receptor 2 (HER2) negative, 14% had HER2 +1, 23% had HER2 +2, and 35% had HER2 +3 (Figure 28). P53 was positive in 38% of the cases (Figure 29). 89% of the studied women had undergone adjuvant chemotherapy, 9% had undergone neoadjuvant chemotherapy, and 2% did not receive any chemotherapy (Figure 30). Most patients (95%) had received external radiotherapy after surgery and a limited number of cases (5%) had received intraoperative radiotherapy (Figure 31). 90% of the patients had only received Tamoxifen for hormone therapy regimen (Figure 32).

**Figure 33:**
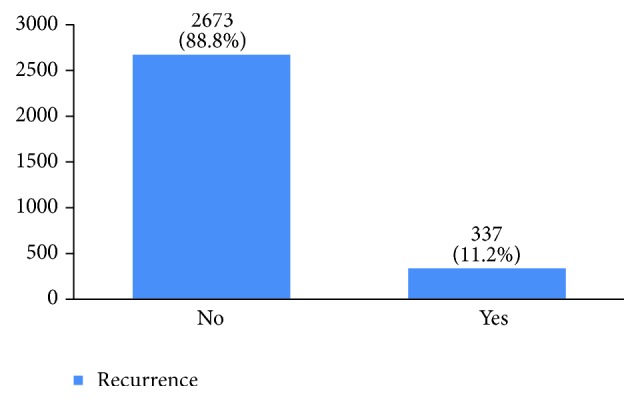
The percentage of recurrence in the studied cases.

**Figure 34:**
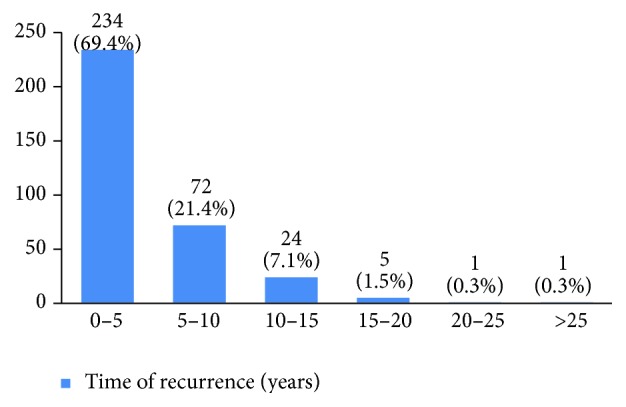
The time of recurrence in the studied cases.

**Figure 35:**
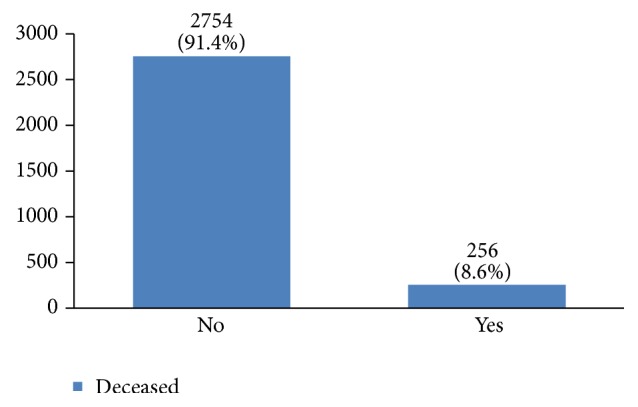
The percentage of the deceased because of breast cancer in the studied cases.

**Figure 36:**
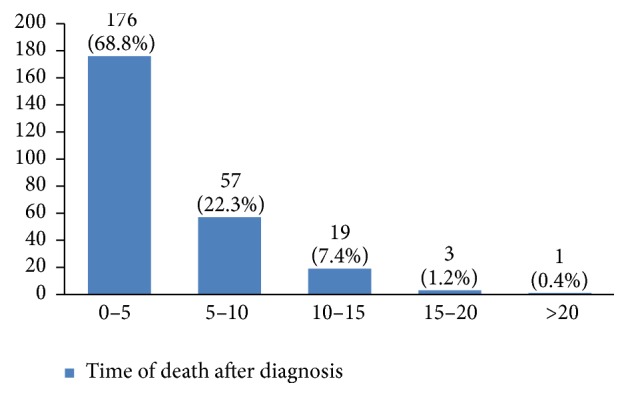
The time of death after breast cancer diagnosis in the studied cases.

Recurrence happened in 11% of the studied women. Most of them (69%) had happened in the first five years of diagnosis (Figures 33 and 34). 8.6% of the patients had died mostly in the first five years after diagnosis (Figures 35 and 36).

## 4. Discussion

The risk of getting breast cancer increases with age and most breast cancers occur in women older than 50 years old [6, 7]. In our study, most women with breast cancer were 40 to 50 years old. This is in accordance with other similar studies, which have stated that breast cancer occurs a decade earlier in Iranian women compared to women of western countries [4, 8–10]. Today, since 46% of Iran's population is 20 to 44 years old (around 19 million women) [11], finding the best screening method for such a big population is the next step for early diagnosis of this disease and improving Iranian women's life quality.

Some studies have found an association between educational status and breast cancer [12]. Our findings show that most Iranian women with breast cancer are educated. Since having medical literacy is an indicator of social status, it is important to investigate the relationship between education and breast cancer in more detail.

Around 94% of our patients were married and according to some studies, being married can have a positive effect on the early diagnosis, treatment, and survival of breast cancer [13, 14]. According to Iran's national statistics, around 70% of Iranian women aged 15 to 65 years old are married.

Around 41% of the studied women had up to two pregnancies and 50% had up to two childbirths. In this regard, it has been shown that multiparity has a preventive effect against breast cancer [3] and nulliparity increases the risk of breast cancer [15–19].

In our study, 30% of the studied women had experienced an abortion, but there is still controversy about the relationship between abortion and breast cancer [20–25]. Also, many studies have found that breastfeeding can decrease the risk of breast cancer occurrence [26–30]. Breastfeeding for less than 24 months is a risk factor of breast cancer [31]. Although there are many recommendations for breastfeeding for at least 24 months in Iran, 12% of the studied women had not breastfed their children. To find out whether the duration of breastfeeding on the whole or for each successful labor is influential on the risk of breast cancer requires further investigation.

Having a positive family history of breast cancer is a risk factor for breast cancer. Familial breast cancer consists of 20–30% of all breast cancer cases [3, 6, 7, 32]. In our study, 18% of patients had first- or second-degree family member(s) with breast cancer.

Hormone replacement therapy increases the risk of breast cancer occurrence [3, 31–35]. 29% of our patients had a history of hormone consumption. More studies are needed for a better understanding of the association between hormone replacement therapy and breast cancer in Iranian women. Increasing fat intake can also increase the risk of breast cancer occurrence [36, 37]. In this regard, 48% of our patients had a high-fat diet. A high-fat diet can result in obesity and increasing the body mass index leads to higher risks of this cancer [38–40].

Smoking can increase the risk of breast cancer occurrence [36, 41]. Unfortunately, the number of women smokers is increasing. According to the Iranian Atlas of Women, 4.3% of Iranian women were smokers in 2004. This has increased to 6.9% in 2010 [11]. In our study, 6.6% of the studied women were smokers. This shows that the percentage of women with breast cancer who are smokers is the same as the general population. Because this study has covered a period from 1998 to 2014, 6.6% smoking rate in Iranian women for the whole period may indicate a higher prevalence of smoking in breast cancer patients.

According to the national assessment of health and diseases, the prevalence of diabetes in cities, villages, and the whole country is 2.9%, 1.2%, and 2.3%, respectively. The high percentage of women with breast cancer who also had diabetes (34%) compared to the general population shows that there might be an association between diabetes and breast cancer and that diabetes might also influence the treatment outcomes of this cancer [42–46].

About 66% of our patients had experienced a natural menopause, while one-third had a history of hysterectomy. It has been shown that there is an association between late menopause and an increased risk of breast cancer [6].

Breast conserving treatment can be done in all stages of breast cancer, unless the breast size does not allow it. 59% of our patients underwent breast conserving treatment. However, this method can be used more often because it has a survival rate similar to modified radical mastectomy and the recurrence rate has also been similar in all types of breast conserving treatments [47–49]. Our data can be a valuable resource to evaluate local and distant recurrences and survival rate of various breast conserving treatments in Iranian patients.

In our study, 55% of the patients were diagnosed with a 2 cm to 5 cm tumor. Because tumor size is an important factor in staging breast cancer, type of treatment, and survival rate [50], developing a national program for increasing women's awareness about the benefits of screening is necessary. More than 10 lymph nodes had been removed in 44% of studied women but only 53% had one or more positive lymph nodes.

Almost half of the tumors had been detected in stage 2. Tumor stage is also an important factor in local and distant recurrences, survival rate, and having an effective early diagnosis program. The regional distribution of stages 3 and 4 of the tumor in Iran should be explored in future studies. More than 50% of tumors were with intermittent grade. Since tumor grading is influential in the treatment outcome [51], further studies should be done with multivariate analysis to determine the effective factors on tumor grading.

Most tumors (87%) were invasive ductal carcinoma which is in agreement with other studies [11, 52, 53]. Because pathology type is related to recurrence and disease-free survival [54], we are very interested to do further studies to find out which factors have a significant association with the type of pathology.

About 55% of our patients had undergone axillary surgery. Because the type of axillary surgery usually depends on breast cancer stage, it shows that most breast cancer patients in our study were diagnosed beyond stage 1. Thus, more screenings are needed for early detection of this disease. Although in some studies the left breast had been more involved, in our study both breasts were involved equally.

Lymphovascular invasion is a predicting factor of tumor metastasis and an important prognostic factor in patients with lymph node-negative and invasive breast cancer [6]. In our study, lymphovascular invasion was found in 42% of the cases. The next step is to investigate interactions between lymphovascular invasion and other factors to predict treatment outcome and recurrence.

Hormone receptor status is a main factor in planning breast cancer treatment. In our study, 70% of breast cancers were positive for estrogen receptor and 67% were positive for progesterone receptor. Breast cancers with HER2 gene amplification or HER2 protein overexpression are called HER2-positive. HER2-positive breast cancers tend to grow more quickly and are more likely to spread and come back compared to HER2-negative breast cancers. HER2 is also a predictive and prognostic factor for breast cancer [6]. Previous studies in Iran have reported a 14% to 71% prevalence of this factor in breast cancer patients, which might be due to the difference in their measurement methods. In this study, HER2 +3 was 35%, which is much more than other countries (10%–15%) [3, 15, 17, 29]. The importance of this factor is that breast cancer patients with HER2 should have their own special treatment, such as Herceptin treatment for HER2 +3. Clinical studies have shown that overexpression of HER2 is associated with a poorly differentiated tumor, high proliferation ability, positive lymph node, less hormone receptor expression [6], and a higher risk of recurrence. To determine whether this was the case with our patients requires further multivariate studies.

About 37.5% of our patients were P53 positive. Several factors, such as estrogen and progesterone receptors, human epidermal growth factor receptor 2 (HER2), age, stage, grade, time since metastatic development, and the site of metastasis, have been well identified as predictive criteria for this cancer's prognosis. P53 is also a well-studied marker in breast cancer, but its significance in predicting clinical outcome remains controversial [32].

Most of our patients (89%) received adjuvant therapy. Neoadjuvant therapy was prescribed for only 9% of the cases. Chemotherapy analysis of early breast cancer showed that this treatment can decrease chances of recurrence and mortality in women younger than 70 years old who have breast cancer at stages 1, 2A, and 2B [6]. Neoadjuvant therapy provides the opportunity to study primary tumor response and metastases to regional lymph nodes to characterize a special regimen.

Radiotherapy is done at all stages of breast cancer whether the patient has undergone breast conserving surgery or modified radical mastectomy. In recent years, intraoperative radiotherapy is done alongside breast conserving surgery. 94.5% of our patients received external radiotherapy and 3% were candidates for intraoperative radiotherapy in the last two years. According to some studies, Herceptin decreases mortality up to 33% and recurrence up to 50% compared to cases that have only received chemotherapy [6]. Only 7% of our studied women had received Herceptin.

The recurrence rate in our study was 11%, with 69% occurring within the first five years after treatment. Breast cancer is the fourth cause of death due to cancer in the world, but it is still a common cause of death due to cancer among women in developing countries. In our study, 8% of the women died due to breast cancer. The burden of this disease is expected to increase in developing countries [55, 56]. The mean five-year survival rate of Iranian women with breast cancer was 71% in 2007 [5] compared to 92% in the US [6]. This figure was 69% in our 16-year period retrospective study.

## 5. Conclusion

Our study revealed original findings that can be used in national policymaking to find the best screening method for early detection and to improve the quality of care and prevention of breast cancer in Iran. It also provided a rich data source for bivariate and multivariate analysis (modeling). We are using this data to develop a balanced score card for recurrence and overall survival for newly diagnosed patients.
